# Valerianate of Zinc

**Published:** 1844-07-27

**Authors:** 


VALERIANATE OF ZINC.

This new preparation is extolled by some of the Italian physicians as a very powerful remedy in several nervous affections. It is formed by adding the protoxide of the metal to the vegetable acid to saturation, and then slowly evaporating the solution. It is administered in the form of pill in the dose of one or two grains. In the *Bulletino delle Sc. Mediche* some cases of neuralgia successfully treated by it are recorded.

